# “I Hope She’ll Feel Happier To Be alive”: A Qualitative Study of Hope among Family Members of Individuals with Mental Illness

**DOI:** 10.1007/s10597-025-01557-7

**Published:** 2025-11-05

**Authors:** Rikke Amalie Agergaard Jensen, Jeanne Holm Ovesen, Jens Peter Eckardt, Elsebeth Stenager

**Affiliations:** 1 Research Unit Mental Health South West, Department of Regional Health Research, University of Southern Denmark, Odense, Denmark; 2 Center for Involvement of Relatives, Mental Healthcare Services, Region of Southern Denmark, Vejle, Denmark; 3 Better Psychiatry, the Danish National Association of Caregivers (Research Unit), Copenhagen, Denmark

**Keywords:** Carer burden, Mental illness, Group-based psychoeducation, Community-based interventions

## Abstract

**Introduction:**

This study explores what family members of individuals with mental illness hope for, and how their hopes are shaped, sustained, or challenged through participation in a group-based psychoeducational intervention program for informal carers. Although hope is a key concept in recovery-oriented practice, little is known about the hopes of carers themselves, or how hope is supported through preventive interventions.

**Method:**

The study draws on qualitative data from a questionnaire completed by 36 family members of individuals with mental illness participating in a group-based psychoeducational intervention program. Of these participants, three were subsequently interviewed according to realist-informed principles. In both cases, data was analyzed thematically.

**Results:**

Family members’ hopes related to their loved one’s recovery, well-being, and societal inclusion, but also to regaining everyday roles, relationships, and identities that had been disrupted by mental illness. Hope was expressed as both a personal and a relational process, situated within wider social and structural contexts. Stories shared within the group played a central role in hope processes, functioning as mirrors (recognizing one’s experiences and feeling reflected in the words of others) and windows (seeing what one could become or might have been) that created perspective and a sense of shared humanity. However, some encounters were experienced as overwhelming or re-traumatizing.

**Conclusion:**

Hope among family members is multifaceted, encompassing personal, relational, and structural dimensions. The findings underscore the importance of programs that actively support carers in sustaining hope as part of the broader recovery context.

Family members, or informal carers, are increasingly recognized as important partners in psychiatric care, with both international and Danish policy frameworks assigning them a central role in treatment processes (Dirk et al., 2017; The Danish Health Authority, 2022). At the same time, growing emphasis is being placed on the idea that treatment for severe mental illness should go beyond symptom management to support the individual’s pursuit of a good and meaningful life. This reflects a broader cultural and paradigmatic shift from seeing individuals with mental illness as passive recipients of care to recognizing them and their families as active agents in recovery (Slade, 2009). This approach is also called personal recovery (Anthony, 1993).

A key concept in this context is hope. The belief that it is possible to fully or partially recover from mental illness and, not least, to move beyond its often devastating consequences (Leamy et al., 2011; Schrank et al., 2008). This hope is not limited to symptom reduction but extends to the possibility of rebuilding a meaningful life despite significant losses, such as shattered dreams, stigma, reduced autonomy, and negative experiences in work life or other social arenas (Thomsen et al., 2023).

Although personal recovery is often described as an individual process driven by the person themselves, recovery is also inherently social. It unfolds in interactions with others, family members, friends, and professionals, who provide support in different ways (Topor et al., 2011). The degree of influence the social network has on the recovery process naturally varies, and it is important to acknowledge that these relationships can also be a contributing factor to the problems the person struggles with (Schön et al., 2009; Topor et al., 2006). Nonetheless, many people who have recovered emphasize the importance of having someone in their life who believed in them and held hope on their behalf when they themselves were unable to do so (Lauzier-Jobin & Houle, 2021; Schrank et al., 2008). In this context, Deegan (1996), a key figure in the user movement, introduced the concept of “surrogate hope,” the idea that others can hold hope for a meaningful and fulfilling life when the person momentarily loses sight of the possibility of light or direction in their life. Related concepts include “vicarious hope” (Topor, 2003) and “substitute hope” (Cullberg, 2007), which refer to offering oneself as an emotional stand-in during times when the person cannot find a way out of their despair. In addition to classic forms of support such as emotional and instrumental help to support hope, mechanisms like presence, communication, and influence have been identified as central (Lauzier-Jobin & Houle, 2021).

Beyond supporting their relative’s recovery, family members themselves can be understood as having recovery journeys of their own, in which hope plays a central role (McCarthy et al., 2023; Poon et al., 2025). Yet, family members’ perspectives are often overlooked in recovery-oriented practice, and their needs are not always recognized (Mekuriaw et al., 2025; Israel et al., 2023). Recognizing family members as active participants in recovery, with processes that are both shared and distinct from those of their ill relatives, and with own individual needs in this, broadens recovery from being an individual trajectory to a family- and system-level phenomenon.

Importantly, family members’ recovery trajectories, like those of service users, are not shaped in isolation but within wider social, institutional, and cultural contexts. This calls for a broader understanding of recovery as not only personal and relational, but also societal. Responsibility does not rest solely with the individual and their close network, but also with the wider structures that shape everyday life (Borg & Kristiansen, 2004; Onken et al., 2002). Recovery-supportive contexts may involve inclusive and enabling practices that allow people to engage in society on their own terms (Borg & Kristiansen, 2004; Redublo et al., 2024). As Spandler and Stickley (2011) argue, there can be no real hope for recovery without the development of supportive social contexts and cultures that actively support it. Other studies likewise emphasize accessibility, responsiveness, and trust as critical contextual factors for strengthening hope (Sælør et al., 2014; Yeung et al., 2020).

Although hope is widely recognized as a key factor in service users’ recovery, less attention has been given to how family members themselves experience hope and what helps them sustain it. This gap is important to address, as family members are often expected to serve as a source of hope when the person they support loses their own. However, carrying hope on behalf of another is not always straightforward (McCarthy et al., 2023). Family members may become so emotionally burdened by the situation and the dynamics of the relationship that even vicarious hope becomes depleted. Family members often experience significant psychosocial strain (Gérain & Zech, 2021), and as psychiatric treatment increasingly shifts to outpatient settings, they often find themselves caught in a dual pressure: managing their own distress while simultaneously taking on greater responsibility for their relative’s recovery journey (Glecia & Li, 2024). Recent work suggests that these challenges should not only be seen in terms of burden, but also as part of carers’ own recovery processes, in which hope plays a central role (Poon et al., 2025). Supporting carers therefore means not only acknowledging their instrumental role in a relative’s recovery, but also recognizing and responding to their personal needs.

Drawing on a carer perspective centered on the narratives and lived experiences of family members, this study explores: (1) what family members of individuals with mental illness hope for, and (2) how encounters with others in similar situations can strengthen their hope and foster more hopeful ways of living. By focusing on the nature of hope and the processes through which hope is sustained among family members, this study contributes to a deeper understanding of their emotional and relational worlds. This knowledge may inform the design of interventions that not only support family members in their roles as carers but also nurture their own recovery.

## Method

### Design

This qualitative study is embedded within a combined process- and outcome-evaluation of a group-based psychoeducational program for carers, carried out in accordance with the Medical Research Council’s framework for complex interventions (Skivington et al., 2021). The evaluation was informed by principles of realist inquiry (Pawson, 2006, 2013), which view outcomes as context-dependent and shaped by underlying mechanisms, and this framing also guided the present study. Ontologically, we recognize reality as stratified and complex, where outcomes emerge from the interaction of mechanisms and context. Epistemologically, knowledge is understood as generated through interpretation of family members’ lived experiences, informing causal explanations through theory development. This approach also accommodates exploration of more phenomenological questions, such as what family members hope for, while maintaining a realist focus on mechanisms (i.e., what works). Collecting data from multiple sources, including written responses and realist-informed interviews, enabled us to construct a nuanced understanding of hope, its manifestations, and the conditions under which it is fostered.

### Setting and Procedures

From August to December 2023, 150 carers participated in a group-based psychoeducational intervention delivered by Bedre Psykiatri (Better Psychiatry), a Danish national civil society organization supporting family members and other informal carers of individuals with mental illness. The program is designed as a structural response to the often invisible burden of caregiving, combining traditional psychoeducational content, such as knowledge about mental disorders, coping strategies, and the healthcare system, with a strong emphasis on carers’ lived experiences, mutual recognition, and peer support. Information about the program can be found in Table 1.

**Table 1:** Overview: A group-based, nation-wide program for informal caregivers of individuals with mental illness (the CarerCourse)

Sessions	Content	Learning objectives
Session 1 (2 h)	Introduction to the group and the program, with overall purpose of aligning expectations.	Participants feel safe and comfortable in the group, and experience that the program aligns with their hopes, values, and expectations.
Session 2 (2 h)	Introduction to the structure of the diagnostic system, including categories of mental disorders and transdiagnostic features. Emphasis on how diagnoses may change over time and how they impact carers’ identity and self-understanding.	Participants gain knowledge about diagnoses, mental illness, and symptoms, and reflect on how this understanding relates to their own reactions and role as caregivers.
Session 3 (1 ½ hours)	Overview of the healthcare system, including the role of general practitioners and common challenges in treatment pathways.	Participants gain insight into psychiatric treatment options and the role of general practitioners, helping them navigate care systems more effectively.
Session 4 (2 h)	Focus on carers’ own experiences, reactions, and coping strategies. Introduction to psychological concepts such as vulnerability models, protective factors, and problem-solving techniques.	Participants develop awareness of common caregiver responses and psychological mechanisms, and learn strategies to strengthen their own mental resilience. Emotions and thoughts are normalized through shared reflection.
Session 5 (1 ½ hours)	Overview of relevant legislation and carers’ rights, including the Social Services Act and the Public Administration Act.	Participants gain practical knowledge of legal frameworks enabling them to better navigate support systems, particularly within municipal services.
Session 6 (2 h)	Summary of key themes from previous sessions and exploration of how participants can sustain the peer network once the program has ended.	Participants learn how to maintain and make use of the group as an ongoing support network where experiences are shared, reflected upon, and drawn on to manage everyday challenges.

The course consists of six sessions, with 16–20 participants per group. Two sessions include a guest presentation by a carer volunteer affiliated with Better Psychiatry, who shares their lived experience and introduces participants to additional local resources. Each course is facilitated by a trained instructor with a background in health care or social work. The instructor introduces key topics through presentations, exercises, and group dialogue, encouraging participants to contribute their own perspectives

The above descriptions are based on the course manual (fall, 2023). The content is continuously updated based on evaluation results

As part of the program’s pilot evaluation, all participants were invited to complete a questionnaire one week before (*N* = 84) and one week after (*N* = 59) the intervention. The questionnaires included closed-ended items on demographics, well-being, burnout, coping, and satisfaction (see also Blinded for review). The follow-up questionnaire repeated these measures and additionally included an open-ended item with the prompt: “What do you hope for the person you care for?” Of the 59 participants who completed the follow-up questionnaire, 36 provided a written response to this question. These written responses formed the primary data for our analysis of family members’ hopes (research question 1).

In addition, three of the 36 participants were interviewed after completing the program. The interviews were informed by Manzano’s (2016) principles of realist interviewing, including the concepts of theory gleaning and teacher–learner cycles, and focused (among other things) on understanding for whom and under what circumstances the program supported hope. Rather than aiming for representativeness, we selected participants whose direct involvement offered rich insights into how the program operated, prioritizing their potential to generate theoretical understanding (Emmel, 2013). Interviews lasted between one and two hours and were transcribed verbatim. These transcripts provided the primary data for our analysis of how encounters with others in similar situations may strengthen family members’ hope and support more hopeful ways of living (research question 2).

### Participants

The 36 participants were primarily women (97.2%) with a mean age of 54 years (range: 34–72 years). All identified as carers of a family member with mental illness—most commonly a child (77.8%), followed by a spouse or partner (13.9%), or a sibling (8.3%). For more than half of the participants (58.4%), mental illness and associated challenges had been present in the family for four years or longer. Participants reported feeling burdened by the situation to a very high degree (30.6%), a high degree (55.6%), or to some degree (13.9%). None reported feeling burdened to a small degree or not at all.

### Analytical Approach

The data corpus consisted of two qualitatively different, yet structurally similar, types of narrative material: written responses to an open-ended survey question and interview transcripts. While the written responses were largely descriptive and closely aligned with the question prompt, the interview transcripts situated family members’ reflections within the broader context of their everyday lives and experiences with the program. These differences were important to consider when selecting appropriate analytical strategies (Popping et al., 2015), given that research questions, methodological choices, and the nature of the data should be aligned (Maxwell, 2008). For these reasons, we divided the data corpus into two distinct datasets: one consisting of written responses, and the other of interview transcripts. Correspondingly, we present the analytical strategies and findings in two parts: The first part of the analysis focused on family members’ written responses and explored what family members of individuals with mental illness hope for. The second part drew on interview transcripts to examine how encounters with others in similar situations can strengthen family members’ hopes and support more hopeful ways of living.

Both analyses were guided by Braun and Clarke’s (2019) principles for reflexive thematic analysis, but, as described in Table 2, differed in accordance with the nature of the two datasets. Common to both strategies, however, was a thematic approach aimed at identifying patterns of meaning in the material. Each analysis was partly theory-driven, as coding was informed by a conceptual focus on hope and recovery, but also inductive, allowing themes to be developed thro the data itself and shape the final structure of the analysis. Following Braun and Clarke’s phases, we began by familiarizing ourselves with both datasets through repeated readings and initial note-taking. We then coded data extracts relevant to each analytical focus, following the two research questions. Next, we organized the codes into preliminary themes, which were then refined, defined, and named through iterative discussion and collaborative reflection. Finally, we wrote up the results, interpreting the themes in relation to the two research questions.

**Table 2:** Overview of the different coding strategies in relation to the research questions

Research questions	Coding strategy
1. What do family members hope for?	Semantic approach: Following Braun and Clarke’s (2006) definition of semantic coding, we developed codes directly from participants’ explicit statements. The aim was to generate a rich thematic overview of what family members concretely hope for, with attention to personal, social, and structural dimensions of recovery.
2. How can meeting others in similar situations strengthen concrete hopes and support a hopeful life?	Latent approach: Using a latent coding strategy (Braun & Clarke, 2006), we explored the underlying ideas, assumptions, and meaning structures that shape how participants understand and narrate hope. Since participants’ hopes are closely tied to their life conditions and the way hope is cultivated through social encounters, we sought to preserve narrative coherence. To this end, we first analyzed each transcript individually before identifying connections across cases—an approach inspired by Bengtsson & Andersen’s (2020) adaptation of thematic narrative analysis (see also Riessman, 2008).

### Ethical Considerations

The Regional Ethics Committee (journal no. S-20232000-66) confirmed that the study did not require formal ethical approval. The study was subsequently reported to the region’s internal research registry (journal no. 23/25502). The study was conducted in accordance with the Declaration of Helsinki, which outlines key ethical principles, including respect for individuals, informed consent and voluntariness, the right to withdraw, and the minimization of harm.

## Results

The following sections present the two analyses that illustrate different dimensions of hope as experienced by family members of individuals with mental illness. The first analysis situates hope in relation to personal, social, and structural aspects of recovery, exploring what family members hope for on behalf of the person they support. The second analysis focuses on hope as a relational and narrative process, examining how stories and shared experiences function as mirrors and windows, offering recognition through reflection and enabling new perspectives by opening onto previously unseen possibilities.

### Part One: What Family Members Hope for

#### Hoping for Well-Being, Joy, and Meaning in Life

When family members describe what they hope for, they express a deeper longing for the person they care for to regain emotional strength, rediscover meaning, and find a life worth living. In this sense, hope is not simply defined by the absence of symptoms, but by the presence of well-being, purpose, and a renewed desire to live. As Karina, whose words inspired the title of this article, puts it:

I just hope she gets a life she’s happy with (…) That she gets through the losses that are constantly in her life because of the disease. That she comes ‘round the other side’ and that she’ll feel happier to be alive. (Karina)

Lars, another participant, shares a similar sentiment, expressing his hope that his son “will one day be able to laugh again and feel joy. That he can find a meaningful life. One that is meaningful for him.”

Alongside these hopes, many family members also express a desire for their loved one to regain greater autonomy—particularly among parents of adult children. They wish for the person to take more control over their lives and become less dependent on them. Gitte, a mother, reflects on this: “I hope that my daughter can have a more normal life with a job, family, etc. (…) I hope she becomes less dependent on me the older she gets (…), so there can be more room to be me” (Gitte). In Gitte’s account, we sense not only the wish for her daughter to find her own path, but also a quieter, parallel hope: that she herself may be able to step back, rediscover her own identity, and gain relief from the ongoing demands of caregiving. It is a hope that holds both love and a longing for freedom—to remain present, and yet also to let go.

#### Hoping To Restore Disrupted Family Roles

The hope of being there and letting go is accompanied by a desire for the illness not to define or dominate the family’s social relationships. Several participants expressed a longing to return to their natural roles within the family—as parent, partner, or sibling—without being confined to the identity of a carer. As Susan put it, she hoped for “a parent-child relationship where we don’t have to be carers and have that role with him.” Her words reflect a recurring theme throughout the material: that recovery is not only the individual’s journey toward restored identity, values, and direction, but also the family’s journey. Family members carry their own hopes: hopes of reclaiming their roles, of restoring mutuality, and of re-establishing a sense of normalcy in everyday life.

#### Hoping for Systemic Support and Social Inclusion

Family members also express hope for greater support from the systems that surround them. They describe a ‘displacement of responsibility’ (Brigitte), as tasks that should fall to professionals are left to families without adequate support, leading to a profound sense of being ‘completely alone’ (Lars). Hanne articulates this burden: “I wish there was someone with more knowledge and insight than me, someone who could help find the best way for [my child] to live. We can’t afford to pay for it ourselves.” In this way, the feeling of being alone also signals deeper structural inequalities, where access to help is uneven and insufficient. As Hanne’s account illustrates, carers not only seek recognition for their role but also hope for a system that actively shares the responsibility and helps carry the weight of care.

The structural dimension of hope also extends to the broader societal level. Here, hope is linked to inclusion, the aspiration that the person with mental illness can find a place in society where they feel valued and are able to contribute with the resources they have. This may involve finding ‘meaningful work’ (Christine) or ‘finishing education’ (Annie). The hope for inclusion also extends to social belonging. Kathrine, for instance, expresses a desire for her daughter to reconnect with youth communities and be seen as more than her illness:

[M]y hope is that she becomes part of youth communities again and can be allowed to live as a young person coping with her loneliness and anorexia. That she experiences that she is taken seriously as a valuable human being. (Kathrine)

### Part Two: Stories and Hope Processes

To understand how stories support hope, we draw on the metaphors of stories as windows and mirrors. When a story functions as a window, family members see what they or their lives could become, but are not yet—or what they might have been, but never became. When a story instead serves as a mirror, family members recognize their experiences reflected in the words of others and feel a sense of mutual recognition.

These different functions shape how stories contribute to hope processes, either by offering perspective or by affirming belonging. As the analysis illustrates, stories rarely function in just one way: they can act as windows, mirrors, or both at once, depending on the dynamics between listener and teller, including the resonance arising through their interaction.

### When Stories Are both Mirrors and Windows

Stories that connect family members with others in similar situations, but with different outcomes, open new ways of understanding what might be possible. They offer alternative perspectives and broaden the horizon of hope. For Nete, such perspective begins to take shape as she listens to another carer describe how, after long struggles, they finally feel supported by the system. In contrast, Nete’s own experience is marked by a lack of recognition and support; as she puts it, she feels that “the system [has] never shown any understanding.”

However, by revealing an alternative path, one that suggests change is possible even after long-term disappointment, the story offers Nete hope that the system might also respond differently in her family’s case. What once felt like a deadlock now opens up a new space for action, prompting her to reflect on her role:

Well, it really set many questions in motion for me, like: ‘How is it that they [the professionals] saw her [the other carer] that way, but not me?’ (…) and I started thinking about how (…) I could use what she had been through for something. I mean, I was thinking, ‘how could I change that [professional’s] mindset?’

For Nete, the story functions both as a mirror, affirming her experience, and as a window, offering her a vision of constructive change. Together, these dual functions cultivate hope by shifting how she perceives herself, her family’s situation, and her capacity to influence it for the better.

### When Stories Are either Mirrors or Windows

Stories that mirror help family members recognize themselves, fostering a sense of connection and belonging. Sanne describes “sitting in a context where I didn’t have to explain much because the person you are talking to knows exactly what you are talking about.” Even Ane, who at first doubts that the other carers have anything to offer, gradually comes to feel less alone. She realizes that her feelings are shared by “people who are in a similar situation”, and this, as she puts it, opens up for her “a place to turn to.”

In a life often marked by alienation, mirroring others and being mirrored offers Ane a brief but powerful sense of being truly known: a rare space of safety, free from judgment or misunderstanding. Here, Ane senses an understanding “that runs on the inner lines”, a kind of shared emotional language between those who have lived through similar experiences. In that space, she feels free to “vent in a different way, because there was no one who doubted or (…) responded [to her experiences] with: ‘Wow – that must be difficult.’”.

For family members, seeing themselves reflected in others can also shift their perspectives, allowing them to observe themselves as if from the outside. During the first session of the program, a carer volunteer who has gained some distance from the most intense phases of caregiving, shares their journey. Despite lacking the energy to hear about other people’s lives, the story evokes recognition for Ane, sparking a sense of belonging that extends beyond the darkest corners of life to include its brighter moments. By seeing herself reflected, Ane realizes that she, too, has moved to a different and better place. Recognizing that life can evolve for the better—even when all hope has long since faded—affirms that the most complex and seemingly intractable aspects of caregiving will eventually loosen:

There was a lot of resonance (…). She [the volunteer] used a phrase like: ‘When I felt really bad and just sank deeper and deeper into that darkness where there’s no light at all—not even when you look up—and when you’re being eaten up by hopelessness and powerlessness.’ I could easily recognize those feelings. But I could also recognize where she is today. Fortunately. And you could say, if nothing else, I could use it to say: ‘I’ve also come further—I’ve also moved on’. (Ane)

In the previous section, we illustrated how stories can function as mirrors reflecting familiar experiences back to family members. Yet stories may also serve as windows, offering a view into lives that differ from their own. In these cases, it is the contrast rather than the similarity that allows family members to reframe their situations. This becomes evident in Sanne’s experience: as she listens to others’ stories, she finds room to come to terms with the conditions that shape her life by focusing on what sets their situations apart from her own:

When you hear others’ stories, then… then you also, shall we say, measure your own against theirs. For me, that’s meant that there were some stories where I thought: ‘Oh, phew—good thing it’s not me, I’d rather have what I have.’ There’s a kind of perspective in that way of participating. Not that it’s a bed of roses, but you might (…) meet someone whose situation is worse. (Sanne)

A quiet sense of hopefulness emerges from coming to terms with one’s circumstances: in realizing that, despite everything, things could have been worse, Sanne finds a renewed belief in life, a sense that it will, somehow, carry on: “I don’t know, [it gives] maybe a little, a little surplus in the energy account, or something… Or that famous ‘never mind’ button, where you [think]: ‘Oh well, okay. It’ll all work out, one way or another.’”.

At first glance, recognizing that others are worse off might signal a downward trajectory or even seem like resignation. But for Sanne, it highlights that suffering is not static, allowing difficulties, burdens, and fears to be understood as dynamic and therefore changeable conditions. By framing her situation as something she would ‘rather have,’ Sanne asserts a measure of agency. In doing so, she repositions herself within a context otherwise marked by powerlessness, enabling her to come to terms with her family’s circumstances—finding balance between changing what can be changed and accepting what cannot.

### Stories and Hopelessness

Although stories are predominantly conducive to family members’ sense of hope, they can also nourish feelings of hopelessness. When this happens, it may be because family members are unable to recognize themselves, their hopes, or expectations in the reality they are faced with. In such situations, the interaction with others and their stories becomes alienating, unsettling, or even disheartening.

Ane recalls past experiences of meeting other carers who were, as she puts it, “out of their minds with anxiety.” While she recognizes traces of her own former suffering in their distress, she also realizes that anxiety no longer holds the same grip on her. Still, the recognition evokes strong emotions, offering Ane a glimpse into a time she thought she had left behind. This revitalizes Ane’s latent trauma, leaving her in emotional chaos. Moreover, when others “take up space,” there is little room left for Ane’s needs. This combination of emotional overload and perceived marginalization leaves her feeling ‘completely abandoned’:

When I entered the room, I was suddenly faced with things I had shut down. And you could say… there’s nothing wrong with that. Because when I looked at them, I thought: ‘Oh my God that was me five years ago.’ But it also made me feel completely abandoned—emotionally. I told [the instructors] that I found it difficult, because they [the anxious participants] were taking up space. They were dying of fear. (Ane)

## Discussion

This study explored the nature of hope and hope processes among family members of individuals with mental illness. The analyses show that carers hold hope not only for the recovery of the person they support, including a return to meaning, autonomy, and joy, but also for the restoration of relationships and identities disrupted by mental illness. Moreover, their hope extends beyond the individual level, encompassing a hope for social inclusion and structural support, both for the person with mental illness and for themselves. In this way, hope emerges as a personal, social, and structural phenomenon. These findings support existing research on recovery processes (Davidson et al., 2016; Leamy et al., 2011; Topor et al., 2011), while also adding an important perspective on family members’ own hopes and positions within these processes (McCarthy et al., 2023). Recent research on carers’ recovery reinforces this point, showing that family members’ own sense of connectedness, identity, and meaning is often fragile, and that recognition of their role and needs is a crucial but frequently overlooked dimension (Poon et al., 2025; Redublo et al., 2024). Our findings also reveal that hope is closely intertwined with grief—grief over lost roles, strained relationships, and the life that might have been. As others have observed (Bland & Darlington, 2002), carers may long for freedom, the return of everyday normalcy, and relief from the responsibilities that caregiving imposes. Central to this is the possibility of reclaiming identities as mother, father, sibling, or partner—roles often eclipsed by the demands of caregiving. When such roles can be re-established, it supports not only carers’ well-being, but also enables the person with mental illness to rebuild a sense of self that is not defined solely by illness. These findings align with earlier research showing that caregiving can be experienced as isolating and emotionally burdensome, and that it can disrupt rather than nurture familial closeness (McCarthy et al., 2023).

In terms of hope processes, our findings show that encounters with others in similar situations can revitalize carers’ sense of hope by opening up new perspectives on how to live hopefully alongside uncertainty. This occurs through the sharing of stories that offer recognition or perspective. Such stories function as relational mechanisms that connect carers and activate a sense of common humanity (Gilbert & Leahy, 2007). Stories can act as mirrors, validating suffering and showing that one is not alone, or as windows, offering alternative ways of coping (Ling et al., 2019). In this way, shared humanity becomes a catalyst for hope, transforming isolation into connection and despair into possibility (Alasiri et al., 2019). Similar mechanisms have been observed in participatory programs based on recovery principles, which strengthen carers’ hope and resilience (Redublo et al., 2024).

In addition to showing what carers concretely hope for and how their hope takes form, this study also portrays hope as a complex, multifaceted phenomenon, dual in more than one sense. On the one hand, we can think of hope as something personal and subjective: it is something that family members carry with them (some more solitary than others), and it can only exist because they trust that the future may hold possibilities, not only suffering. On the other hand, hope also reaches beyond the individual. It is hope for the loved one, but also for oneself. It is a hope that society will recognize, support, and include both the person with mental illness and those who stand beside them. At once inward and outward, hope is an act of individual trust that takes shape in what is shared and human—and is shaped, in turn, by the institutions, communities, and systems that surround and respond to the family. This mirrors understandings of hope as a relational and moral stance (Mattingly, 2010; Topor et al., 2011), in which personal longing is sustained, or thwarted, by the social and structural conditions in which it unfolds. Our results therefore echo the call for a more relational and inclusive recovery paradigm that does not only center service users but also acknowledges and supports carers’ parallel journeys (Zhou et al., 2025; Poon et al., 2025).

### Implications for Practice

The findings presented above show that family members’ hope is not merely a private or psychological matter but also a public concern. As a society, we not only have a stake in this hope, but a responsibility to ensure that carers can live hopefully, even amid the psychosocial strains many of them endure.

Programs, such as the one evaluated, can be seen as one such response to this responsibility. Through its group-based format and empowering ethos, it offers a model for how carers might be brought into connection with one another, enabling experiences of shared humanity and mutual support. In doing so, it illustrates how structural interventions can help sustain carers’ capacity for hope—both for themselves and for the person they support. At the same time, the study shows that not all encounters are hopeful. For some, the group setting may stir unresolved grief or reactivate past trauma, leading to emotional overwhelm. These findings suggest that while peer-based spaces can be powerful, they are not universally beneficial.

At a practice-near level, professionals can strengthen carers’ hope by recognizing them as more than supporters—listening to their perspectives, validating their experiences, and affirming their roles. Everyday encounters should leave space for hope and future perspectives, not only symptoms or burdens. Such conversations may help carers to reclaim aspects of identity and meaning beyond the caregiving role.

Finally, the findings suggest that carers themselves can actively cultivate hope not only by focusing on the recovery of their relative but also by legitimizing their own hopes for restored relationships, for balance in everyday life, and for personal wellbeing. Encouraging carers to seek supportive networks, whether through peers, communities, or professional services, may provide vital spaces for sustaining hope when family relationships are strained or ambivalent because of the consequences of mental illness.

### Limitations

This study is based on data from participants enrolled in one particular program, which may limit the generalizability of the findings. The participants’ experiences may not reflect those of all carers of people with mental illness, particularly individuals who do not have access to similar support programs. Moreover, all participants in this study identified as close family members. As such, the perspectives of informal carers who are not family, such as friends, neighbors, or more peripheral supporters, remain unexplored. In addition, the study does not distinguish between different types of family relationships (e.g., parents, partners, or siblings), which may shape both the content of hope and the dynamics of caregiving in important ways. Finally, our analysis focused on carers’ peer interactions and did not examine in depth the relationship between carers and their relatives, a dimension that may also shape how hope is experienced.

### Conclusion and Future Directions

Hope among family members is multifaceted, encompassing personal, relational, and structural dimensions. The findings underscore the importance of programs that actively support carers in sustaining hope as part of the broader recovery context. Future studies might explore how hope processes vary across care systems, cultural backgrounds, and forms of support, as well as how relational positioning and changes over time shape the capacity to hope.

## Data Availability

The data that support the findings of this study are not publicly available due to privacy and ethical restrictions in accordance with the General Data Protection Regulation (GDPR), as the qualitative nature of the material makes full anonymization impossible.
